# Vaccine Inequities, Intellectual Property Rights and Pathologies of Power in the Global Response to COVID-19

**DOI:** 10.34172/ijhpm.2021.57

**Published:** 2021-05-26

**Authors:** Eivind Engebretsen, Ole Petter Ottersen

**Affiliations:** ^1^Centre for Sustainable Healthcare Education, Faculty of Medicine, University of Oslo, Oslo, Norway.; ^2^University of Oslo, Oslo, Norway.; ^3^Karolinska Institutet, Stockholm, Sweden.

 The accelerated development of multiple coronavirus disease 2019 (COVID-19) vaccines is unprecedented and the result of a unique collaborative effort between industry, public health agencies and university laboratories.

However, the introduction of new vaccines has also raised concerns about how the vaccines will be made available in sufficient quantities and distributed fairly across the globe.^1^ In October 2020, Eswatini, India, Kenya, and South Africa proposed a waiver from certain provisions of the World Trade Organization’s Agreement on Trade-Related Aspects of Intellectual Property Rights (TRIPS) that would allow poor countries to produce their own vaccine.^2^ However, the waiver has been met with suspicion especially from high-income countries, and the European Union (EU), the United Kingdom, the United States and several other nations have opposed the proposal.

Using the EU statement^3^ as a case in point, we will demonstrate how a critical analysis of the arguments used to oppose the waiver reveals what Paul Farmer^4^ has referred to as the pathologies of power in global health: global health policies sometimes undermine their own premises by implicitly increasing global inequities rather than reducing them. As Farmer argues, global health policies can lead to “structural violence” by propagating social arrangements that “put individuals and populations in harm’s way” by preventing them from meeting their basic needs.^5^ Farmer has primarily analyzed structural violence in terms of social and economic arrangements. However, as we will argue below, this violence also has a discursive aspect in the sense that economic and social arrangements are propagated and euphemized through language and arguments.^6^

In their response to the waiver, the EU shares the overall ambition of “equitable access to vaccines across the globe,” and they emphasize that this ambition also includes “developing countries” (EUs terminology) that have “no production capacities.”^3^ The “lack of manufacturing capacity” in “developing countries” is thus considered as a given and static fact: the EU implies, as we read it, that equitable access to vaccines across the globe is required *because* poor countries are assumed to be incapable of producing vaccines themselves. The possibility that *increased* production capacity in these countries might be a way forward for obtaining more “equitable access” is thus excluded from the outset.

Accordingly, the EU claims that “there is no indication that IPR (Intellectual Property Rights) issues have been a genuine barrier in relation to COVID-19-related medicines and technologies.”^3^ Rather they emphasize that the real barrier is to be found in the “lack of manufacturing capacity” in poor countries. Through this statement they seem to imply that the real barrier is rooted *within* these countries rather than in international reward systems. Measures to tackle “current and future supply-side shortages” must, in their view, be found outside the IPR system and include “broad and equitable global distribution,” as well as removal of “unnecessary barriers to trade, abolishing tariffs on pharmaceutical and medical goods.”^3^

Although the EU assures that they are committed to work with all members to achieve equitable access to vaccines, they underscore that researchers and pharmaceutical industry have put “extraordinary efforts” into the vaccine development and that their contributions therefore deserve particular support.^3^ This is where the EU defines the real purpose of a well-functioning IPR system: IPR are intended to ensure that extraordinary efforts are “adequately incentivized and rewarded.”^3^ The IPR system is therefore “part of the solution rather than an obstacle” by being “one of the main economic incentives” to stimulate great achievements.^3^ Poor countries that allegedly have no manufacturing capacities are literally left behind; their efforts are not incentivized nor rewarded. Furthermore, the emphasis on economic incentives does not reflect the fact that the unprecedented efforts of pharmaceutical industry to develop new vaccines have been firmly anchored in state funded research.^7^

As their final argument, the EU states that global collaboration is “the *only* way to overcome a global pandemic” [Our emphasis].^3^ As we understand the text, global collaboration, being the “only way,” is here contrasted with local solutions. Hence, “global collaboration” is used as an argument against the global right to vaccine production^3^: solutions should be sought in globally coordinated projects and, by consequence, not in the development of local production facilities in poor countries.

What are the ideologies behind these arguments? A premise for the EU response to the TRIPS waiver is, according to out interpretation, a specific idea of globalization in which the global is defined from the point of view of the fittest (ie, rich countries). To the EU, global collaboration means adapting to a global vision or effort, rather than opening up to a global *diversity* of perspectives and approaches. ‘Global collaboration’ does not only refer to the acknowledgment of global interdependency as a fact but also as a norm^6^: the EU implicitly attributes global legitimacy to their own set of standards and procedures.

This global gaze is also an averted gaze.^4^ As the French philosopher Jean-Luc Nancy^8^ has claimed, the transformation of the global from fact to norm implies termination of the “global” as a plurality of opinions and meanings: “It suffices to say that a worldview is indeed the end of the world of views, the latter being sucked up, absorbed and dissolved in one unified vision.”^8^ Hence, this worldview which underpins EUs arguments implies turning a blind eye to the world of views but also to the structural violence that is characteristic of the neoliberal era: the logic of the market.^4^ A similar logic has been detected in the arguments used against the distribution of antiviral treatment in low-income areas in the early phase of the AIDS epidemic. The neoliberal language of cost-effectiveness was used as a cover-up for structural violence and injustice and ended up “looking more like strategies for managing rather than challenging, inequality.”^4^ Another example is the lack of global incentives to facilitate the production of an effective vaccine against Ebola, an epidemic that existed in poor areas for several decades before a successful vaccine was introduced.

Implicit in the EU arguments is a similar ideology: a competition-driven market model is the text’s pathology of power. The EU’s argument assumes that global collaboration is something that has its center in rich countries and is conditioned by the Western market, and not something that can happen on the terms of poor countries, which are considered to have “no production capacities.”^3^

This leads to a paradox: Lack of efficiency and capacity in the health service in poor countries is used as an argument for globally defined measures, and against contributing to the development of capacity and improving efficiency by allowing these countries to develop vaccines and treatment programs themselves.^9^ Underpinning this paradoxical argument is a static notion where change and improvement are not envisioned. In contrast to the anticipatory gaze characteristic of Agenda 2030, the waiver response does not look beyond the problems of the present. The “lack of manufacturing capacity”^3^ in poor countries is taken to be a timeless fact and the response thereby belies the need for change that Agenda 2030 strongly holds up for us. Decisions taken during this pandemic should prepare for the next. The presentism underpinning the EU response is at odds with universal preparedness for health that the world so strongly needs.^10^

Although other countries that opposed the waiver were less explicit about their reasons, they supported the same line of argument as the EU. Without developing the argument further, the UK described the waiver as an extreme measure to address an unproven problem and as potentially “counterproductive.”^11^ Norway stated, on their hand, that the existing agreement already reflects the “required balance” between “incentives for the development of new medicines and medical products” and “the need for national flexibilities to make exceptions in extreme situations.”^12^ The United States have until recently supported the same argument. However, following an open letter^13^ in which 170 former heads of state and Nobel laureates called on President Biden to support the waiver, the president recently announced that he would share the “know-how” on vaccine manufacturing.^14^ The result of this statement still remains to be seen.

We do not claim that changing the IPR regulations will alone solve the problem of vaccine inequity. There might be other hurdles such as trade secrets or tacit know-how that might be difficult to articulate and share^15^ and other solutions and innovations to address vaccine inequity are needed. A case in point is the mRNA vaccine technology transfer hub recently launched by the World Health Organization (WHO) inviting proposals to scale up manufacturing of mRNA vaccines in low- and middle-income countries.^16^

However, the discussion about the TRIPS waiver is about much more than intellectual property rights. It concerns fundamental ideologies and values in global health. As Jeffery Sachs pointed out in a recent commentary: “Given the surge of COVID-19 in several regions, most recently in India, the continuing emergence of new and deadly variants of the virus, and the inability of the current vaccine producers to keep pace with global needs, an intellectual property waiver or its equivalent has become a practical urgent need as well as a moral imperative.”^17^

TRIPS shows how “economic power can shape global rule making, with far-reaching consequences for health” and put the whole edifice of global health to the test^18^ If we fail to distribute vaccines fairly we risk cementing poor-rich dichotomies and inequities rather than alleviating them – in stark contrast to the goals set out in Agenda 2030.

## Ethical issues

 Not applicable.

## Competing interests

 Authors declare that they have no competing interests.

## Authors’ contributions

 EE and OPO conceptualised the paper. EE wrote the first draft; OPO revised and edited the paper; EE and OPO agreed on final draft.
